# Cesarean Hysterectomy in Abnormally Invasive Placenta: The Role of Prenatal Diagnosis

**DOI:** 10.3390/diseases9030056

**Published:** 2021-08-17

**Authors:** Ana Maria Cubo, Ana Villalba Yarza, Irene Gastaca, María Victoria Lapresa-Alcalde, Maria José Doyague, Cristina Gónzalez, José María Sayagués

**Affiliations:** 1 Department of Obstetrics and Gynecology, Salamanca University Hospital and IBSAL, Paseo San Vicente 58-182, 37007 Salamanca, Spain; avillalba@saludcastillayleon.es (A.V.Y.); igastaca@saludcastillayleon.es (I.G.); mvlapresa@saludcastillayleon.es (M.V.L.-A.); mjdoyague@saludcastillayleon.es (M.J.D.); 2 Department of Pathology, Salamanca University Hospital and IBSAL, Paseo San Vicente 58-182, 37007 Salamanca, Spain; cgvelasco@saludcastillayleon.es (C.G.); ppmari@usal.es (J.M.S.)

**Keywords:** abnormally invasive placenta, cesarean section, myometrial invasion, hysterectomy, prenatal diagnosis

## Abstract

An abnormally invasive placenta (AIP) is a placenta that cannot be removed spontaneously or manually without causing severe bleeding. It is a dangerous condition associated with a high rate of maternal and perinatal morbidity and mortality due to the high rate of massive bleeding and visceral injuries. The standardized ultrasound diagnostic criteria have helped improve its early diagnosis, which is essential to plan coordinated actions to reduce associated morbimortality. We present a case report in which ultrasound diagnosis played a decisive role, enabling the coordination of a multidisciplinary team and improving the immediate care of both mother and newborn. Cesarean hysterectomy was performed with minimal blood loss and a good postsurgical recovery.

## 1. Introduction

Abnormally invasive placenta (AIP) is a placenta that cannot be removed spontaneously or manually without causing severe bleeding. Depending on the myometrial invasion, it can be classified as placenta accreta, increta, or percreta [1,2]. AIP is a dangerous condition, since it is associated with high maternal and perinatal morbidity and mortality rates secondary to massive bleeding and visceral lesions when an emergency cesarean section is performed. The estimated incidence varies from 1/2500 to 1/500 [1,3], although it is increasing since the main predisposing factor is the existence of placenta previa associated with a previous cesarean section [2,3,4]. This is one of the reasons, among others, why WHO is encouraging countries to reduce cesarean rates worldwide [5]. Other risk factors described are anterior placenta previa alone, previous myomectomy, uterine curettage, maternal age over 40, and multiparity [3,4].

Attempts have been made to get AIP early diagnosis. One of the main limitations to achieve it has been the heterogeneous terminology used to define similar issues, which hampers procedure standardization. Another is the lack of controlled studies to draw significant conclusions [1,3]. In 2016, the European Working Group on Abnormally Invasive Placenta (EWG-AIP, which evolved to International Society of Abnormally Invasive Placenta in 2017), through a systematic review and meta-analysis, tried to unify criteria and stated a clinical approach for this condition [3,6].

Early ultrasound diagnosis has shown to improve survival rates and reduce associated complications, including massive transfusions, coagulopathy, or stays in Intensive Care Units (ICUs) [7,8,9]. It is also proven that the coordination of a multidisciplinary team is essential for successful management [1,9,10].

We present the case of a pregnant woman with an AIP. Early ultrasound diagnosis made possible a multidisciplinary and optimal management. Cesarean hysterectomy was performed with minimal blood loss and a good postsurgical recovery.

## 2. Case Report

**Figure 1:**
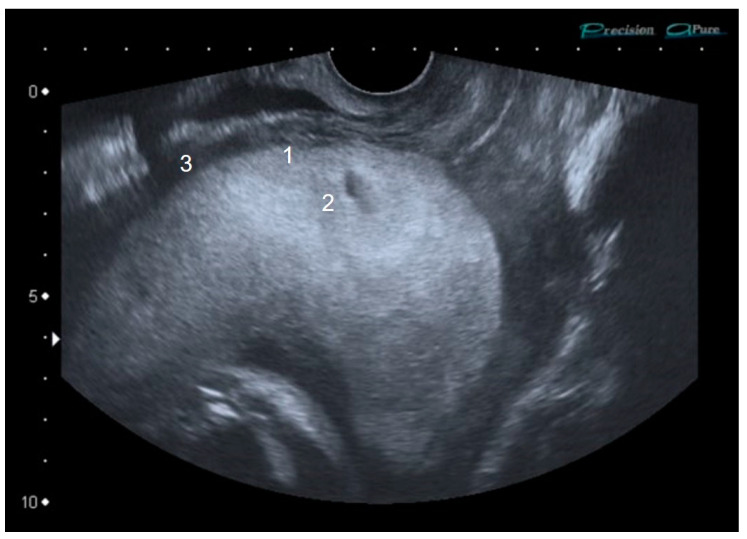
Ultrasound image. Placenta previa at 33 weeks of gestation. (1) The clear zone cannot be visualized. (2) Scarce placental lacunae. (3) Myometrial thinning.

**Figure 2:**
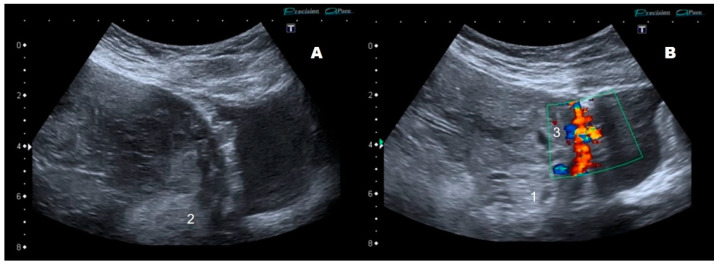
Ultrasound image (**A**): grey scale mode; (**B**): Doppler color mode. Placenta previa at 35 weeks of gestation. Ultrasound signs of placental accretism: (1) presence of placental lacunae, (2) myometrial thinning and loss of the clear zone, (3) increased retroplacental vascularization and bridging vessels to bladder wall.

**Figure 3:**
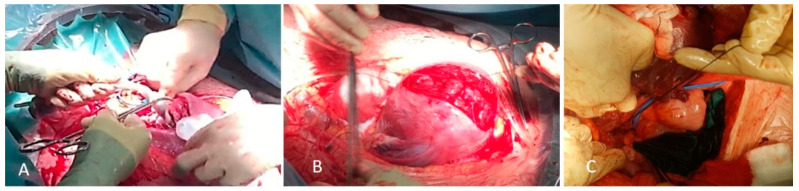
Cesarean section. (**A**) Fundal incision for fetal extraction. Cord clamping, leaving placenta in situ. (**B**) Histerotomy closure, leaving placenta inside the uterus. (**C**) Left hypogastric artery ligation. Left ureter is identified (blue marker).

**Figure 4:**
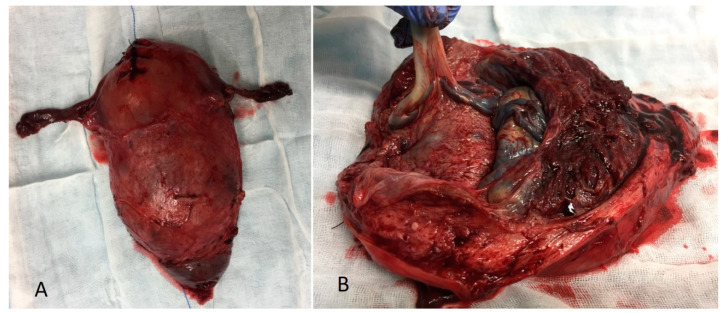
(**A**) Total hysterectomy surgical specimen. (**B**) Opened surgical specimen showing the extension of the placental accretism. Abnormal invasive placenta lays on the whole inferior side of the uterus.

We report the case of a 33-year-old pregnant woman, in her second pregnancy, with a cesarean delivery two years ago. In the second trimester ultrasound, a total, central, occlusive placenta previa is observed, not meeting the International Society of Ultrasound in Obstetrics and Gynecology’s (ISUOG) abnormally invasive placenta (AIP) criteria at that time. Nevertheless, considering the high risk of AIP, appointments were scheduled every 4 weeks. The patient remained asymptomatic. At the 33rd week, a loss of “clear zone”, i.e., loss of the hypoecogenic plane in the myometrium below the placental bed, as well as a myometrial thinning, was identified (Figure 1). At the 35th week, pathological placental lacunae, interruption of the bladder wall without placental bulging, and an increase in subplacental and uterovesical vascularity, as well as the presence of uterovesical bridging vessels were identified (Figure 2). A multidisciplinary team (two anesthesiologists, one obstetrician, one oncological gynecologist, two pediatricians, one hematologist, one urologist, nursing staff, and operating room assistants) was coordinated. A cesarean hysterectomy was programmed at 37th weeks. The patient was informed about the protocol to be followed and signed the informed consent. The massive transfusion protocol was activated and blood and coagulation factors were left ready in case of a massive bleeding event. ICU was advised upon possible admission. Surgery was performed with general anesthesia. A middle infraumbilical incision was made, the abdominal wall was opened, and the uterus was completely exposed, confirming the presence of a central accreta placenta previa. A fundal cesarean section was performed, with a longitudinal incision in the uterine fundus, avoiding contact with the placenta (Figure 3A). A male fetus weighing 3400 g was born. He needed neonatal ventilation due to maternal anesthesia and was admitted to the Neonatology Ward. The placental face cord was sutured and hysterotomy was closed, leaving the placenta inside the uterus (Figure 3B). A total hysterectomy was performed using a LigaSure^TM^ (Valleylab, Boulder, CO, USA) vessel sealing device to reduce the risk of bleeding. Before starting the procedure, both ureters were identified and marked to minimize the chance of ureteral injury (Figure 3C). Hypogastric arteries were identified and ligated (Figure 3C). No invasion of the bladder wall was identified despite the bridging vessels described in ultrasound scan. Bladder integrity was confirmed by the urologist. The total hysterectomy was performed successfully (Figure 4A). Quantified blood loss did not exceed 600 cc, so neither transfusion nor supplementation with coagulation factors were required. Blood pressure and heart rate were kept at normal range throughout the whole procedure.

**Figure 5:**
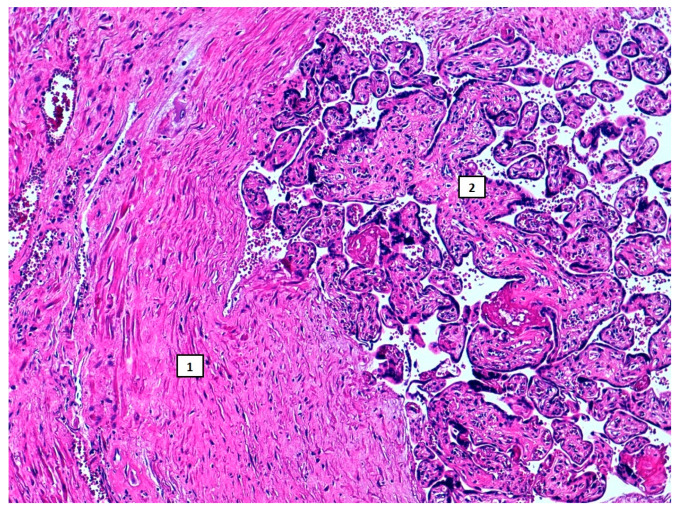
10× magnification hematoxylin/eosin slide. (1) Myometrium. (2) Chorionic villi. Villi lay directly on the myometrium, without intermediate chorionic plaque, a placenta accreta feature.

The surgical specimen showed the extension of the placental accretism, occupying the entire lower face of the uterus (Figure 4B). It was sent to the Pathology Department, AIP diagnosis being confirmed (Figure 5).

During the postoperative period, no bleeding or fever were reported. Both the patient and the newborn were discharged on the third postoperative day.

## 3. Discussion

Abnormally invasive placenta (AIP) is an obstetrical emergency. Its early diagnosis improves perinatal outcomes, as complications can be anticipated by an adequate multidisciplinary approach.

AIP ultrasound criteria have been multiple and varied, as some signs were described under different names, and, in other cases, the same term was used to describe different findings. In 2016, the EWG-AIP published a consensus document [6] in which descriptions of the diagnostic ultrasound AIP markers were unified and standardized [11] in order to create a scoring system to quantify risk [12,13,14].

Our patient had seven of the 10 criteria; loss of the clear zone, myometrial thinning, abnormal placental lacunae, bladder wall interruption, uterovesical hypervascularity, subplacental hypervascularity, and bridging vessels. These signs only began to become evident from the 33rd week onwards. The first two to be seen were the loss of the clear zone and myometrial thinning, whilst the others appeared in the following weeks. No specific order of appearance of ultrasound signs identifying AIP is described in the literature, so their onset is probably not related to either the grade of AIP or its severity [15]. In a meta-analysis by Pagani et al. [16], the signs that showed greater sensitivity for the diagnosis of placenta accreta/increta were the loss of the clear zone (79.6%), the appearance of placental lacunae (74.5%), and lacunar flow (82%). Regarding the moment of ultrasound signs to appear, some authors state that they may be found from stages as early as the first trimester of pregnancy [17]; however, their study is retrospective and analyzes cases with AIP diagnosis confirmed by pathology, so the reported sensitivity would probably be lower if it could be done prospectively.

Considering that AIP is a condition that may progress during pregnancy, ultrasound signs may not be evident at a given time and may be so later. These signs should be sought systematically in patients at risk on the 20th week anomaly scan. The patient should be scheduled every 4 weeks from the 28th week onwards, as they can become more evident in the third trimester [3]. Though MRI has been proved to have a good diagnostic performance in detecting AIP [3,18], it is usually reserved for cases with inclusive ultrasound assessment. In this case, it was not performed, as we considered that ultrasound markers were clear enough to reach a diagnosis. Similarly, 3D and 4D ultrasound are proposed as complementary diagnostic tools. However, it is necessary to have extensive experience in both the performance and interpretation of the exam in order to improve the diagnostic accuracy of 2D ultrasound [19]. This was neither performed in our case.

Although the optimal gestational age for delivery has not been established, the limits reported in the literature for asymptomatic patients range from 34 to 36 weeks [1,2,8,9]. Our patient was managed on an outpatient basis until admission at 37th week, though she was advised of warning signs such as uterine contractions and vaginal bleeding. The decision to wait until week 37 was taken considering the patient was asymptomatic and the best perinatal results are achieved with gestational ages closer to fetal maturity, though we assume that waiting until after the 36th week can also increase the risk of complications.

Regarding birth planning, the decision must be individualized [1,2]. If the patient considers her childbearing desire fulfilled with the current gestation, a cesarean hysterectomy seems to be the most reasonable option [1,2,9]. On the other hand, in cases where the patient expresses a desire to preserve the uterus, conservative techniques may be considered [2,20,21]. These techniques can also be worthy when an uncontrollable hemorrhage is foreseen or if neighboring organ injuries are expected handling a placenta accreta in extreme emergency situations, since they allow the definitive surgery to be deferred until the patient’s hemodynamic situation is better and a dedicated surgical team is located. However, these techniques are not innocuous and a close control is needed, as secondary complications including severe hemorrhage requiring emergent hysterectomy, sepsis, and even pulmonary embolism have been described [20,22]. The only certainty in both the conservative and non-conservative procedures is not to attempt to remove the adherent placenta, as this significantly increases the risk of massive hemorrhage [20,21].

Prophylactic uterine artery embolization has been proposed to minimize blood loss during the surgery in this condition. However, literature results are contradictory, as some authors find a higher rate of operative and postoperative complications [23] whilst others describe better perinatal results with less blood loss [24]. This procedure was not performed in this case.

In the case we are presenting, as the patient already had a previous pregnancy, a cesarean section hysterectomy was chosen. Fetal extraction was performed through a fundal incision. Then, the umbilical cord was clamped and cut leaving the placenta in situ and closing the hysterotomy, performing the hysterectomy afterwards [9,25,26].

The type of hysterectomy performed must be individualized considering the place where the placenta is located, the invasion depth, and the skills of the operating team. Total hysterectomy must be performed in cases of cervical invasion [2,9]. Subtotal hysterectomy can be considered when cervical resection is difficult because of anatomical changes due to placental invasion, though there is no evidence that the use of a subtotal hysterectomy instead of total hysterectomy reduces maternal morbidity or mortality [2,27]. In our case, a total hysterectomy was performed by the team oncological surgeon. Vascular dissection was carried out using a vessel sealing device (LigaSure™, Covidien; Brentwood Blvd Suite 300, St. Louis, MO, USA), minimizing blood loss [28].

Although we are aware of the limited evidence derived from case reports, through this case, we want to highlight the importance of prenatal diagnosis in the management of a life-threatening issue such as AIP. In this case, early diagnosis enabled the coordination of an experienced multidisciplinary team, optimizing resources to give the patient the best care. However, we cannot ignore that, even with an accurate diagnosis and carefully prepared surgery, a worse outcome with significantly higher blood loss is a realistic possibility, and that, though 2D ultrasound is a valuable tool to diagnose AIP, it may, unfortunately, fail to achieve a correct assessment.

## 4. Conclusions

AIP is a life-threatening condition, which can lead to a massive blood loss, endangering mother’s life. Ultrasound criteria unification proposed by the EWG-AIP helps prenatal diagnosis to predict placental invasion and to plan delivery in the most favorable way for both mother and fetus. A multidisciplinary approach using dedicated and trained teams is key to achieving optimal perinatal results.

## Data Availability

Not applicable.

## References

[1] Chantraine F., Langhoff-Roos J. (2013). Abnormally Invasive Placenta—AIP. Awareness and pro-active management is necessary. Acta Obs. Gynecol. Scand..

[2] Collins S.L., Alemdar B., van Beekhuizen H.J., Bertholdt C., Braun T., Calda P., Delorme P., Duvekot J., Gronbeck L., Kayem G. (2019). Evidence-based guidelines for the management of abnormally invasive placenta: Recommendations from the International Society for Abnormally Invasive Placenta. Am. J. Obs. Gynecol..

[3] D’Antonio F., Palacios-Jaraquemada J., Lim P.S., Forlani F., Lanzone A., Timor-Tritsch I., Cali G. (2016). Counseling in fetal medicine: Evidence-based answers to clinical questions on morbidly adherent placenta. Ultrasound Obs. Gynecol..

[4] Jauniaux E., Chantraine F., Silver R.M., Langhoff-Roos J. (2018). FIGO consensus guidelines on placenta accreta spectrum disorders: Epidemiology. Int. J. Gynecol. Obs..

[5] WHO, HRPWHO (2015). Statement on Caesarean Section Rates.

[6] Collins S.L., Ashcroft A., Braun T., Calda P., Langhoff-Roos J., Morel O., Stefanovic V., Tutschek B., Chantraine F. (2016). European Working Group on Abnormally Invasive Placenta (EW-AIP). Proposal for standardized ultrasound descriptors of abnormally invasive placenta (AIP). Ultrasound Obs. Gynecol..

[7] Maher N., Gleeson N., Darcy T., Byrne B. (2016). Comparison of blood transfusion and surgical complications in peripartum hysterectomy when anticipated and unanticipated. J. Obs. Gynaecol..

[8] Warshak C.R., Ramos G.A., Eskander R., Benirschke K., Saenz C.C., Kelly T.F., Moore T.R., Resnik R. (2010). Effect of Predelivery Diagnosis in 99 Consecutive Cases of Placenta Accreta. Obs. Gynecol..

[9] Allen L., Jauniaux E., Hobson S., Papillon-Smith J., Belfort M.A. (2018). FIGO consensus guidelines on placenta accreta spectrum disorders: Nonconservative surgical management. Int. J. Gynecol. Obs..

[10] Shamshirsaz A.A., Fox K.A., Erfani H., Clark S.L., Salmanian B., Baker B.W., Coburn M., Shamshirsaz A.A., Bateni Z.H., Espinoza J. (2017). Multidisciplinary team learning in the management of the morbidly adherent placenta: Outcome improvements over time. Am. J. Obs. Gynecol..

[11] Alfirevic Z., Tang A.W., Collins S.L., Palacios-Jaraquemada R.J. (2016). Pro forma for ultrasound reporting in suspected abnormally invasive placenta (AIP): An international consensus. Ultrasound Obs. Gynecol..

[12] Tovbin J., Melcer Y., Shor S., Pekar-Zlotin M., Mendlovic S., Svirsky R., Maymon R. (2016). Prediction of morbidly adherent placenta using a scoring system. Ultrasound Obs. Gynecol..

[13] Rac M.W.F., Dashe J.S., Wells C.E., Moschos E., McIntire D.D., Wickler D.M.T. (2015). Affiliations expand. Ultrasound predictors of placental invasion: The Placenta Accreta Index. Am. J. Obs. Gynecol..

[14] Gilboa Y., Spira M., Mazaki-Tovi S., Schiff E., Sivan E., Achiron R. (2015). A novel sonographic scoring system for antenatal risk assessment of obstetric complications in suspected morbidly adherent placenta. J. Ultrasound Med..

[15] Jauniaux E., Collins S.L., Jurkovic D., Burton G.J. (2016). Accreta placentation: A systematic review of prenatal ultrasound imaging and grading of villous invasiveness. Am. J. Obstet. Gynecol..

[16] Pagani G., Cali G., Acharya G., Trisch I.T., Palacios-Jaraquemada J., Familiari A., Buca D., Manzoli L., Flacco M.E., Fanfani F. (2018). Diagnostic accuracy of ultrasound in detecting the severity of abnormally invasive placentation: A systematic review and meta-analysis. Acta Obs. Gynecol. Scand..

[17] Cali G., Timor-Trisch I.E., Palacios-Jaraquemada J., Monteaugudo A., Forlani F., Minneci G., Foti F., Buca D., Familiari A., Scambia G. (2018). Hanges in ultrasonography indicators of abnormally invasive placenta during pregnancy. Int. J. Gynecol. Obs..

[18] Familiari A., Liberati M., Lim P., Pagani G., Cali G., Buca Manzoli D., Flacco L., Scambia M.E., D’antonio G. (2018). Diagnostic accuracy of magnetic resonance imaging in detecting the severity of abnormal invasive placenta: A systematic review and meta-analysis. Acta Obs. Gynecol. Scand..

[19] Aryananda R.A., Akbar A., Wardhana M.P., Gumilar K.E., Wicaksono B., Ernawati E., Sulistyono A., Aditiawarman A., Joewono H.T., Dachlan E.G. (2019). New three-dimensional/four-dimensional volume rendering imaging software for detecting the abnormally invasive placenta. J. Clin. Ultrasound.

[20] Sentilhes L., Kayem G., Chandraharan E., Palacios-Jaraquemada J., Jauniaux E. (2018). FIGO consensus guidelines on placenta accreta spectrum disorders: Conservative management. Int. J. Gynecol. Obs..

[21] Matsuzaki S., Yoshino K., Endo M., Kakigano A., Takiuchi T., Kimura T. (2018). Conservative management of placenta percreta. Int. J. Gynecol. Obs..

[22] Fox K.A., Shamshirsaz A.A., Carusi D., Secord A.A., Lee P., Turan O.M., Huls C., Abuhamad A., Simhan H., Barton J. (2015). Conservative management of morbidly adherent placenta: Expert review. Am. J. Obs. Gynecol..

[23] Mohr-Sasson A., Hochman R., Anteby M., Spira M., Castel E., Hendler I., Mazaki-Tovi S., Sivan E. (2020). Cesarean delivery with and without uterine artery embolization for the management of placenta accreta spectrum disorder—A comparative study. Acta Obs. Gynecol Scand..

[24] Yuan Q., Jin Y., Chen L., Ling L., Bai X.M. (2020). Prophylactic uterine artery embolization during cesarean delivery for placenta previa complicated by placenta accreta. Int. J. Gynecol Obs..

[25] Matsubara S., Kuwata T., Usui R., Watanabe T., Izumi A., Ohkuchi A., Suzuki M., Nakata M. (2013). Important surgical measures and techniques at cesarean hysterectomy for placenta previa accreta. Acta Obs. Gynecol. Scand..

[26] Stanleigh J., Michaeli J., Armon S., Khatib F., Zuckerman B., Shaya M., Ioscovitch A., Shenfeld O., Greenblat D., Farkash R. (2019). Maternal and neonatal outcomes following a proactive peripartum multidisciplinary management protocol for placenta creta spectrum as compared to the urgent delivery. Eur. J. Obs. Gynecol. Reprod. Biol..

[27] Tam Tam K.B., Dozier J., Martin J.N. (2012). Approaches to reduce urinary tract injury during management of placenta accreta, increta, and percreta: A systematic review. J. Matern. Neonatal Med..

[28] Gulino F.A., Frigerio L., Bogani G., Rapisarda A.M., Gulino F.A., Frigerio L. (2015). Usefulness of vessel-sealing devices for peripartum hysterectomy: A retrospective cohort study. Updates Surg..

